# Supplementary material for: Microfluidic-spinning construction of black-phosphorus-hybrid microfibres for non-woven fabrics toward a high energy density flexible supercapacitor
Source: Nat Commun. 2018 Nov 1;9:4573. doi: 10.1038/s41467-018-06914-7 (PMC6212570; doi:10.1038/s41467-018-06914-7)
Supplement: Supplementary file 1 — Supplementary Information [file 41467_2018_6914_MOESM1_ESM.pdf]

Microfluidic-spinning construction of black-phosphorus-hybrid microfibers for non-woven fabrics toward high energy density flexible supercapacitors

Xingjiang Wu[a], Yijun Xu[b], Ying Hu[c], Guan Wu[a], Hengyang Cheng[a], Qiang Yu[b], Kai Zhang[b], Wei Chen[b] & Su Chen[a]

[a]State Key Laboratory of Materials-Oriented Chemical Engineering, College of Chemical Engineering, Jiangsu Key Laboratory of Fine Chemicals and Functional Polymer Materials, Nanjing Tech University, Nanjing 210009, P. R. China

[b]i-Lab, Suzhou Institute of Nano-tech and Nano-bionics, Chinese Academy of Sciences, Suzhou 215123, P. R. China.

[c]Institute of Industry and Equipment Technology, Hefei University of Technology, Hefei, Anhui 230009, P. R. China.

Corresponding author: chensu@njtech.edu.cn; gwu2016@njtech.edu.cn; kzhang2015@sinano.ac.cn

Supplementary Table 1 | The pore size distribution and contribution to SSA.

	Pore size distribution (nm)		Mean pore diameter (nm)	$SSA_{micro-pores}$ (m^2g^{-1})	$SSA_{meso,macro-pores}$ (m^2g^{-1})	Total SSA (m^2g^{-1})
	Micro-pores	Meso, macro-pores				
CNTs/BP	0.56~1.04	1.21~95.28	6.28	11.66	67.53	79.19
CNTs/BP-CNTs	0.54~1.09	1.21~95.28	5.27	27.01 (**131.6%** ↑)	82.45 (**22.1%** ↑)	109.46 (**38.2%** ↑)

Supplementary Table 2 | EIS molding data. Parameter values from curve-fitting of the impedance results shown in Figure 3g by using the equivalent circuit described in inset of Figure 3g, where R_0, C_1/R_1, Z_w, and C_2 represent the inner resistance of SCs, contact impedance, diffusion impedance and ion intercalation capacitance, respectively. τ_0 is the relaxation time, also called RC time constant.

	R_0/Ω	C_1/mF $s^{n_1 -1}$	n_1	R_1/Ω	Z_w/Ω	C_2/F	n_2	τ_0/s
CNTs	5.56	0.32	0.68	0.71	49.27	0.79	0.72	9.35
CNTs/BP	5.34	0.28	0.82	1.22	38.34	1.62	0.85	7.14
CNTs/BP-CNTs	5.00	0.31	0.90	0.83	23.75	2.54	0.88	4.95

Supplementary Table 3 | Energy density values of our SC compared with previously reported flexible SCs.

	Electrode materials	Energy density (mWh cm^{-3})	Reference
1	MoS_2-rGO/MWCNTs	1.6	[1]
2	CNTs	2	[2]
3	rGO	2.5	[3]
4	Black Phosphorus	2.5	[4]
5	CNTs/rGO	6.3	[5]
6	MnO_2/carbon cloth	8.3	[6]
7	carbon nanosheet	8.4	[7]
8	graphene/PANI	8.8	[8]
9	Co_3O_4/$Co(OH)_2$	9.4	[9]
10	Ni@MnO_2	11.1	[10]
11	MXene/Graphene	32.6	[11]
12	Li thin-film battery	4V/500 µAh	[12]
13	Our work	**96.5**	

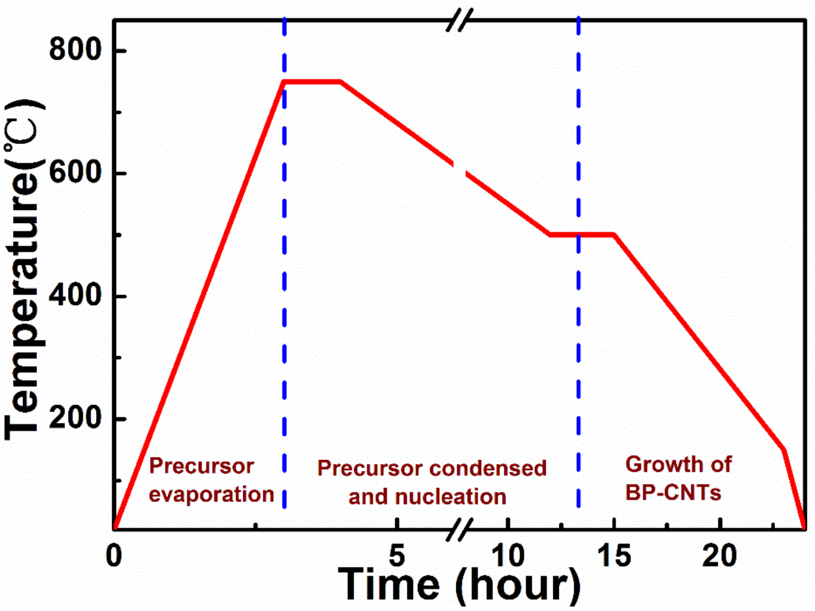

Supplementary Figure 1 | Process of preparation P-CNTs through thermal treatment process.

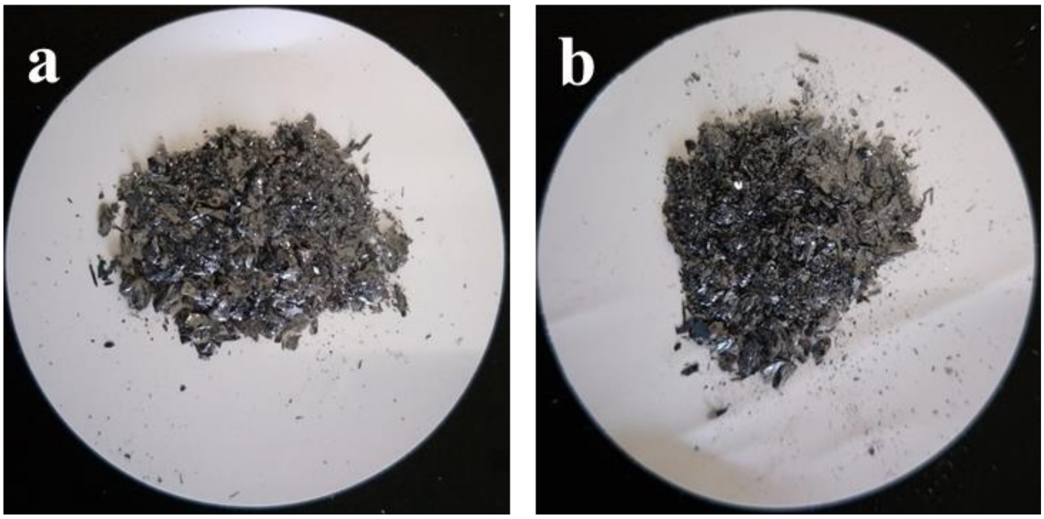

Supplementary Figure 2 | Photograph of BP-CNTs (a) initial synthesis and (b) placed in the air condition for more than one month.

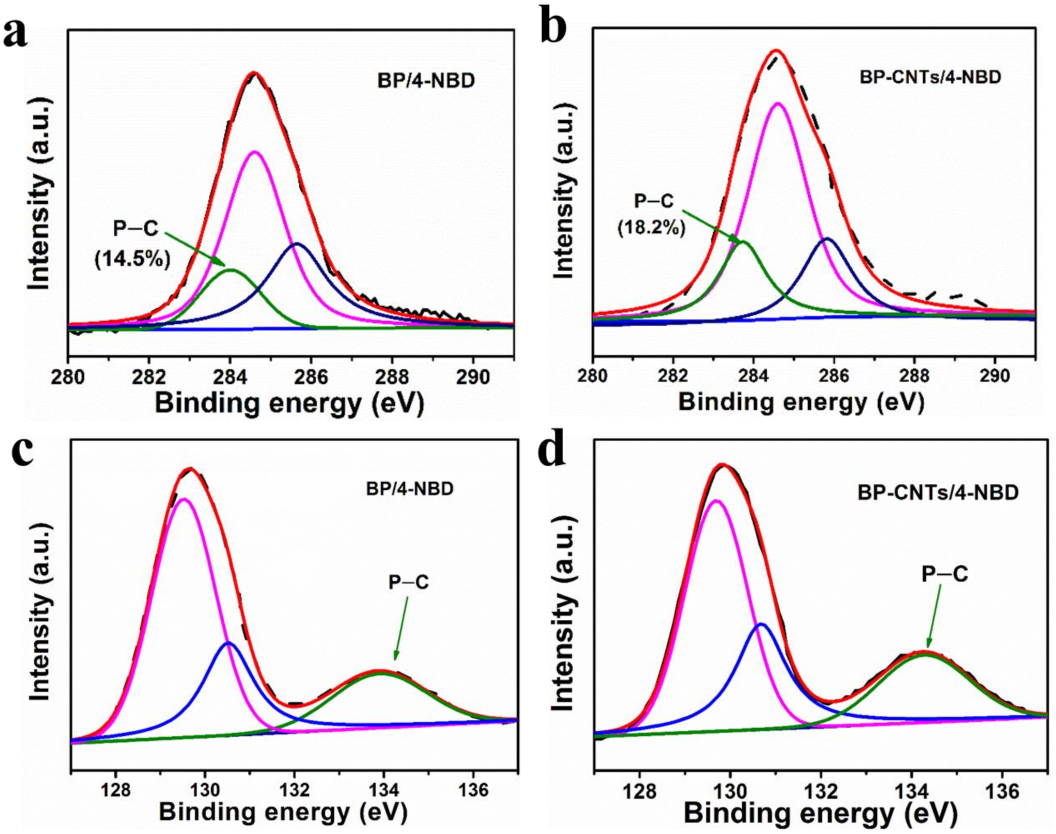

Supplementary Figure 3 | High resolution C1s and P2p XPS spectra of BP/4-NBD (a, c) and BP-CNTs/4-NBD (b, d).

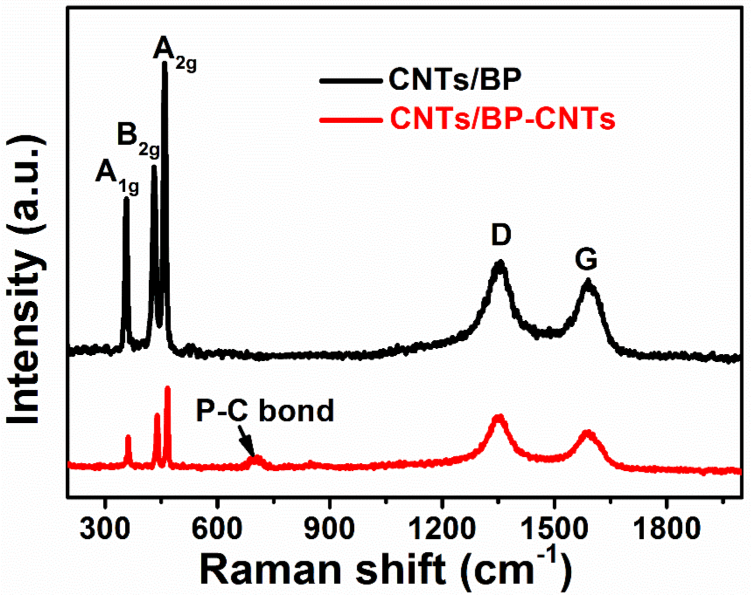

Supplementary Figure 4 | Raman spectra of CNTs/BP-CNTs and CNTs/BP.

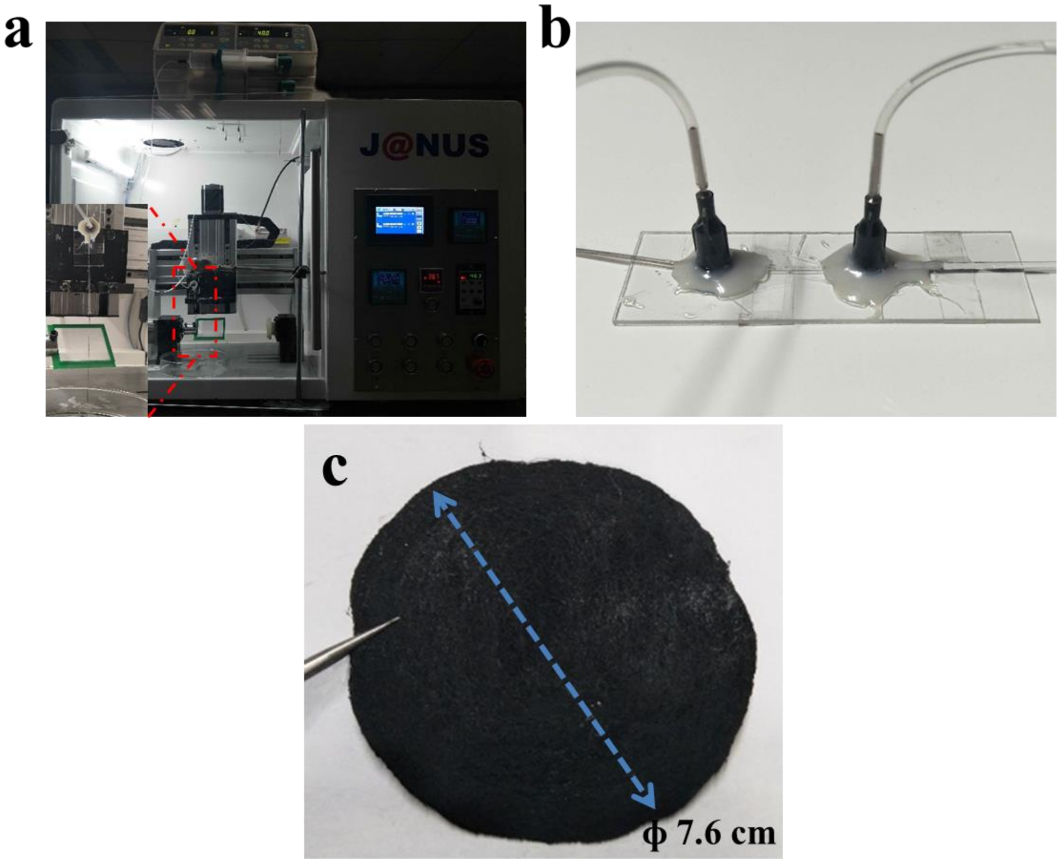

Supplementary Figure 5 | MST fabrication of composite microfiber (a) and the related microreactor (b). Photograph of no-woven fiber fabric (c).

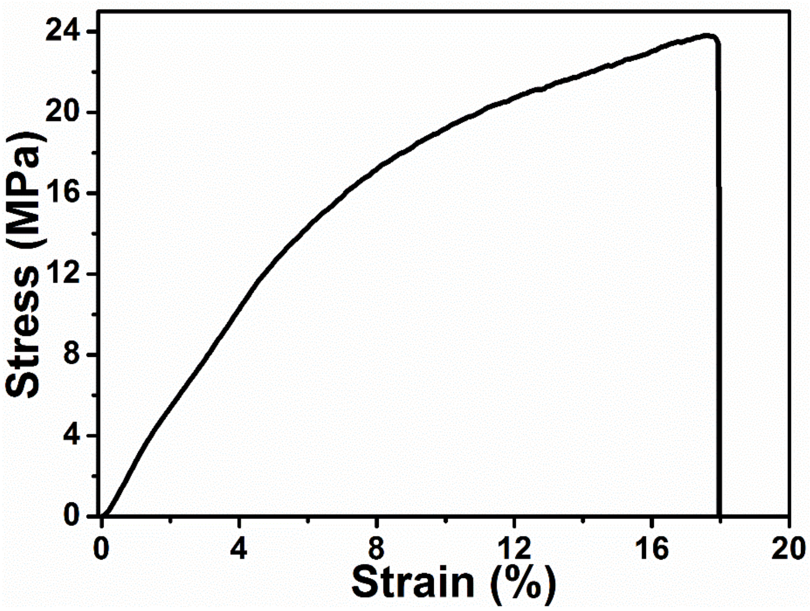

Supplementary Figure 6 | The strain-stress curve of no-woven fabric.

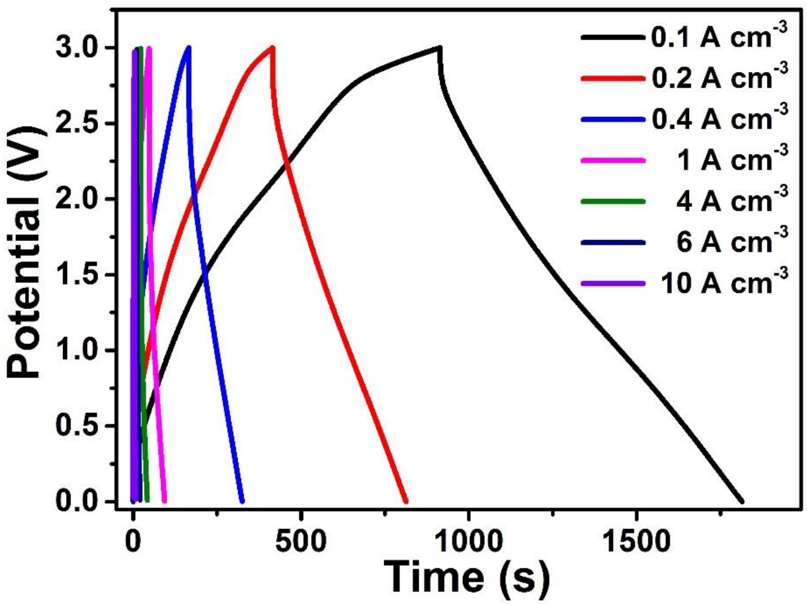

Supplementary Figure 7 | Galvanostatic charge/discharge curve of pure CNTs.

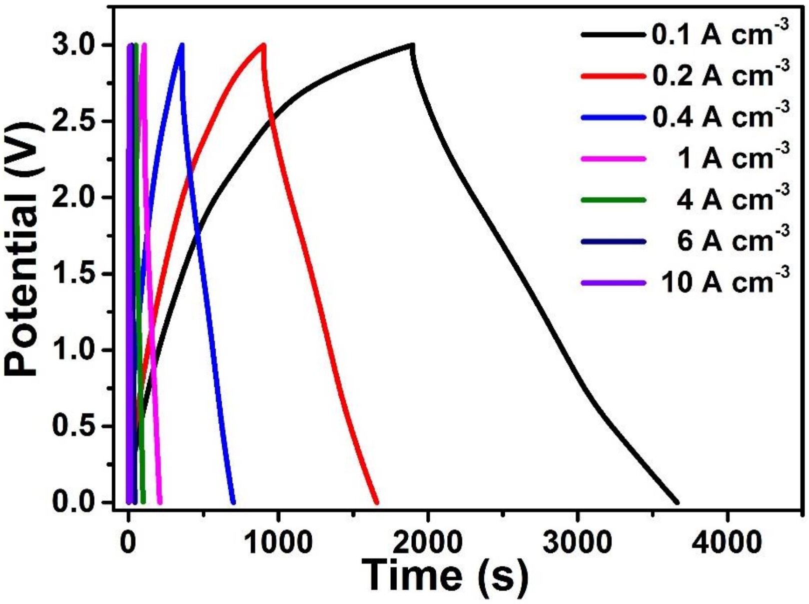

Supplementary Figure 8 | Galvanostatic charge/discharge curve of pure CNTs/BP.

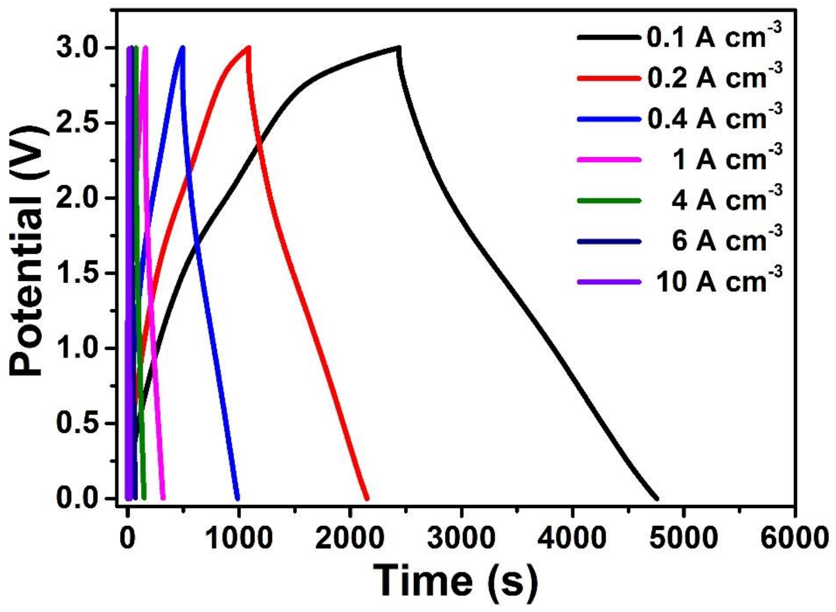

Supplementary Figure 9 | Galvanostatic charge/discharge curve of pure CNTs/BP-CNTs.

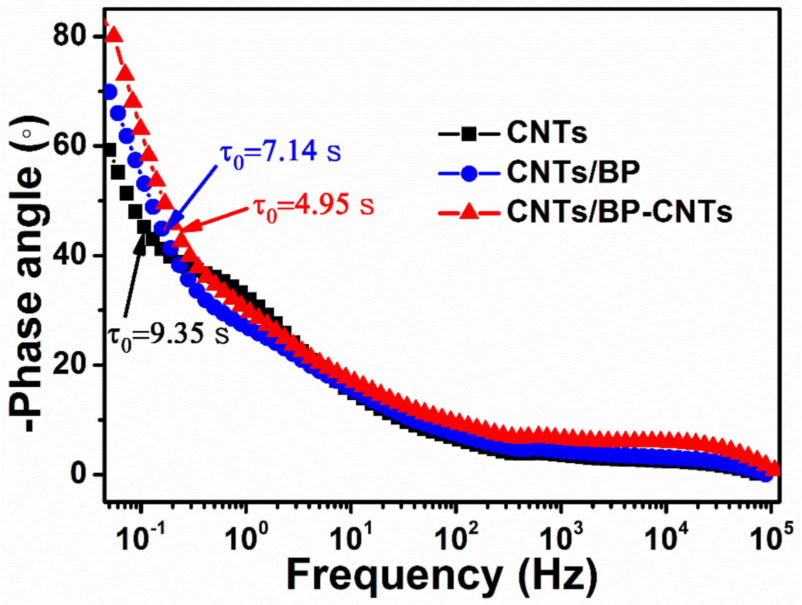

Supplementary Figure 10 | Bode plots of Phase angle vs frequency curves.

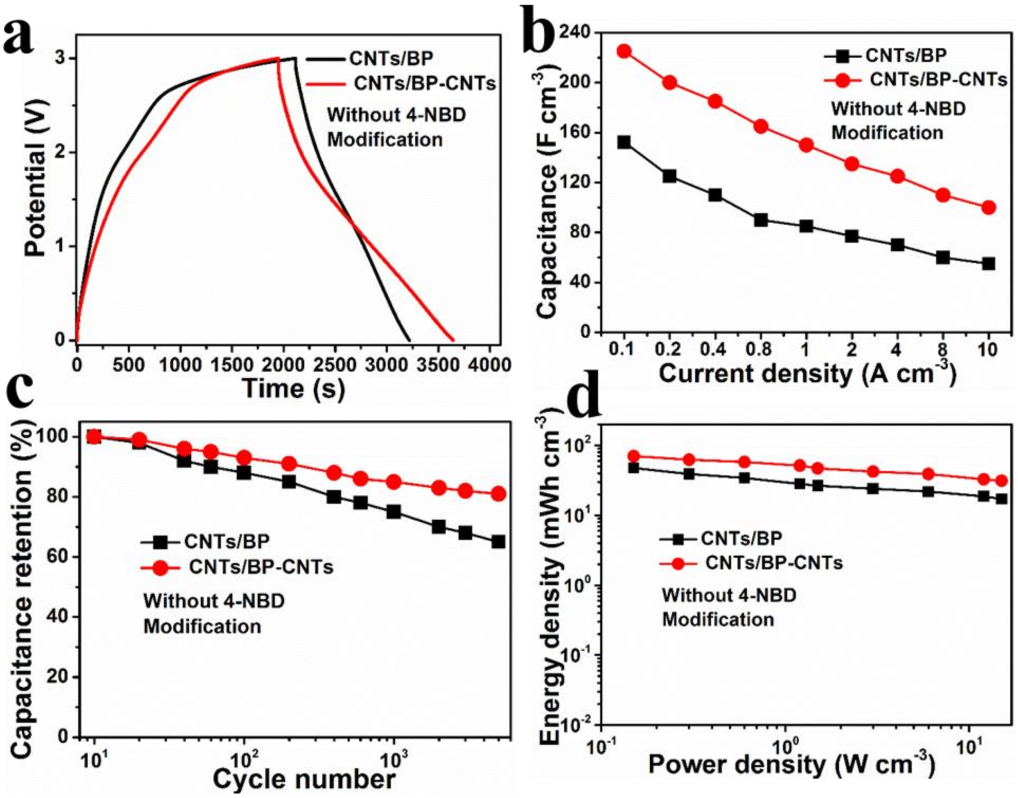

Supplementary Figure 11 | (a) Galvanostatic charge/discharge curves of CNTs/BP and CNTs/BP-CNTs without 4-NBD modification at the current density of 0.1 A cm^{-3}. (b) The calculated specific capacitances under different current densities. (c) Cycle stability of SCs under a voltage of 3 V at a current density of 0.4 A cm^{-3}. (d) Energy density versus power density of SCs.

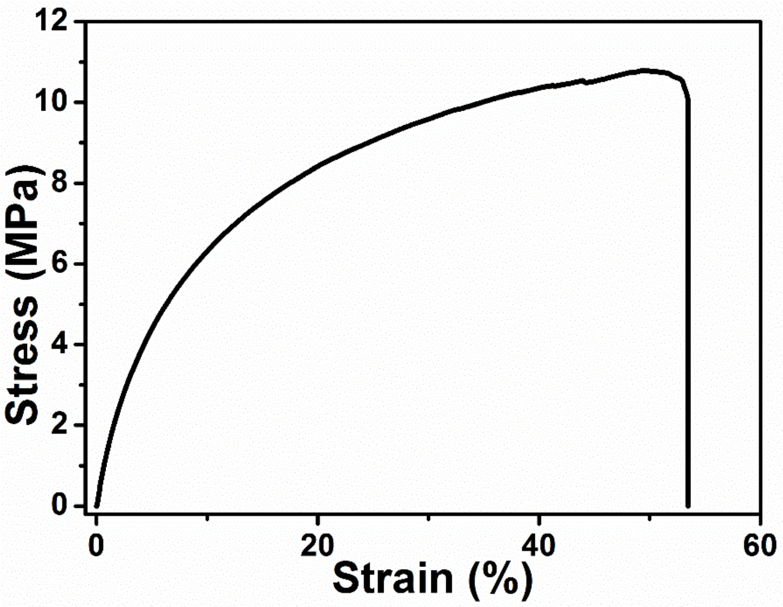

Supplementary Figure 12 | The strain-stress curve of flexible SC.

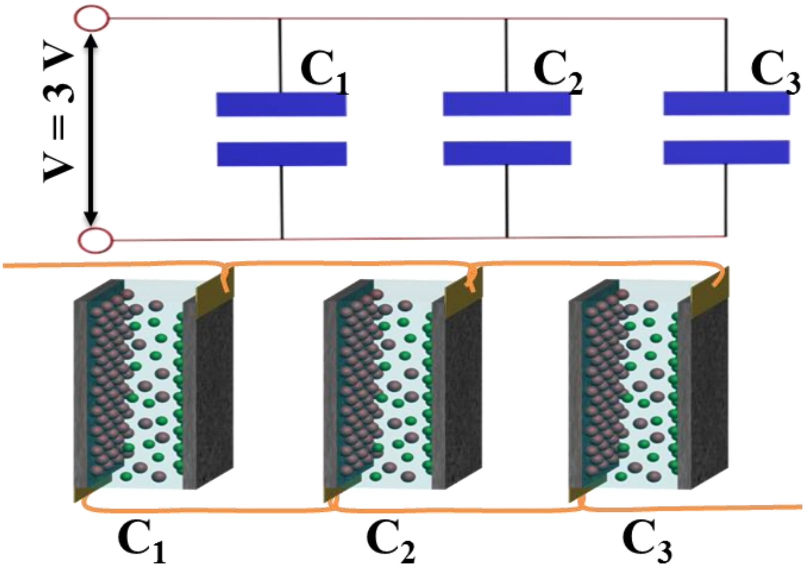

Supplementary Figure 13 | Schematic and equivalent circuit of three SCs connected in parallel.

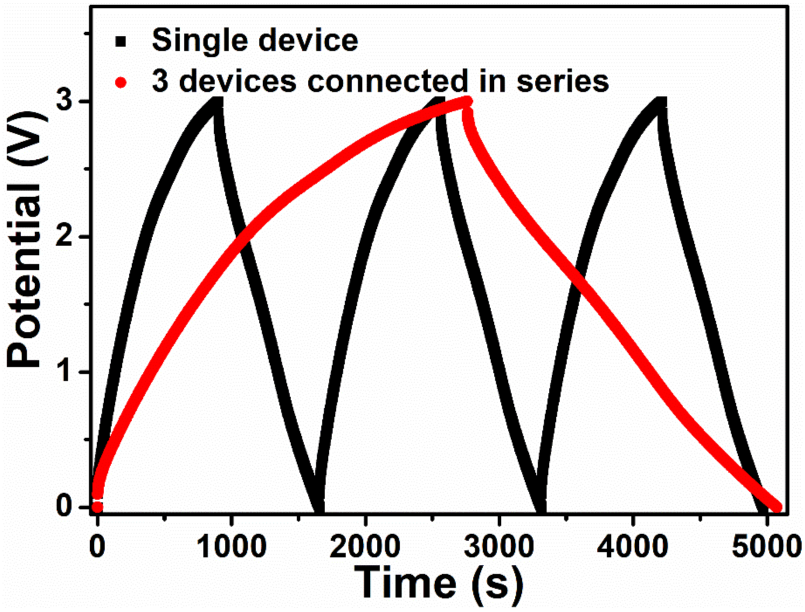

Supplementary Figure 14 | Galvanostatic charge/discharge curves of three SCs connected in parallel.

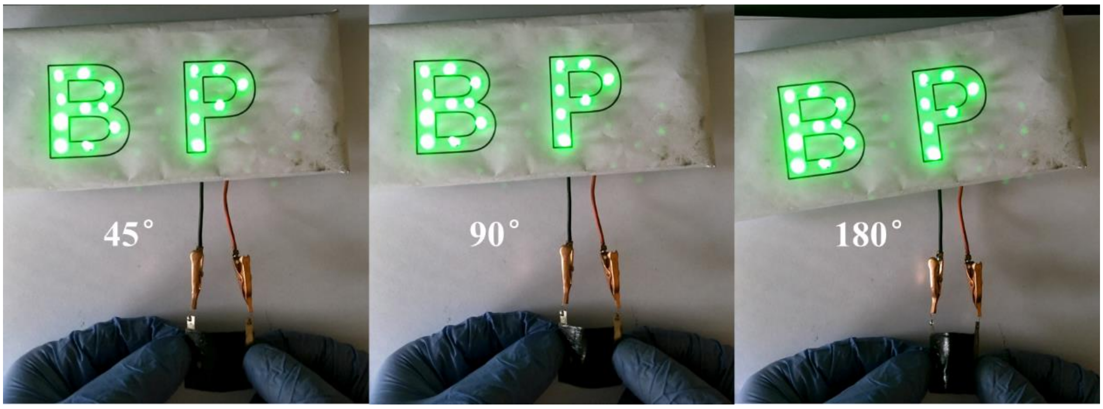

Supplementary Figure 15 | Photographs of flexible SC stably lighting up LEDs under different bending angels.

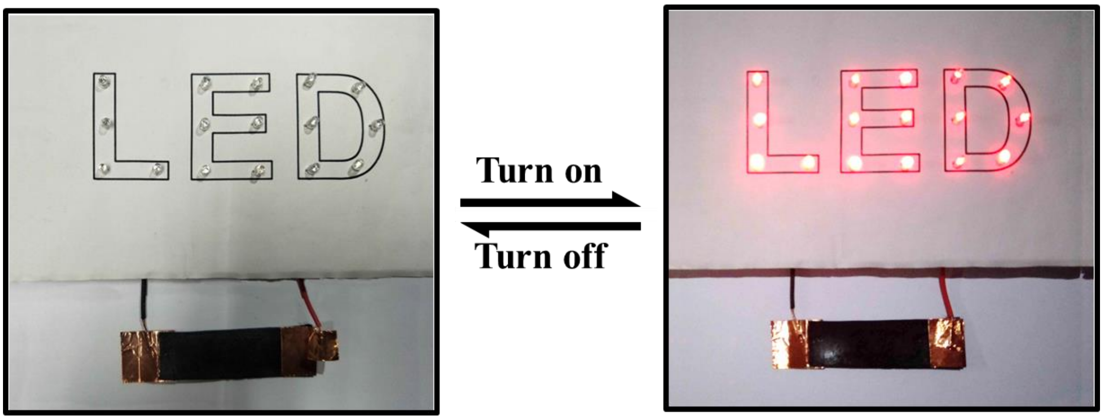

Supplementary Figure 16 | Photographs of flexible SC stably lighting up LEDs.

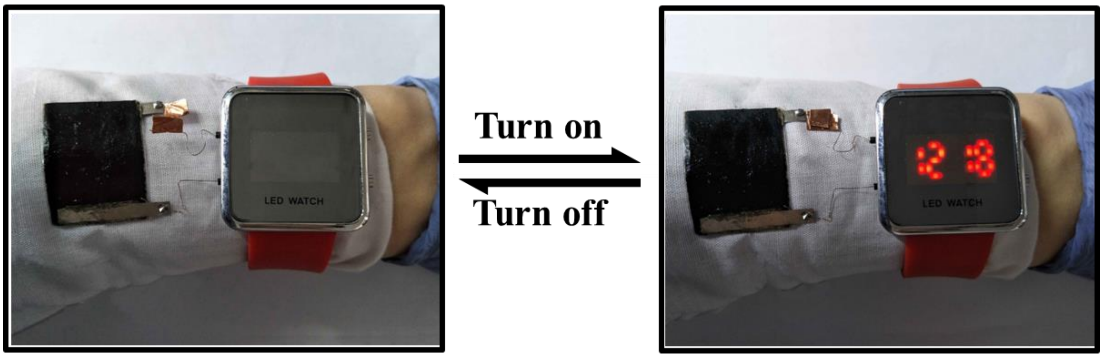

Supplementary Figure 17 | Photographs of flexible SC powering watch.

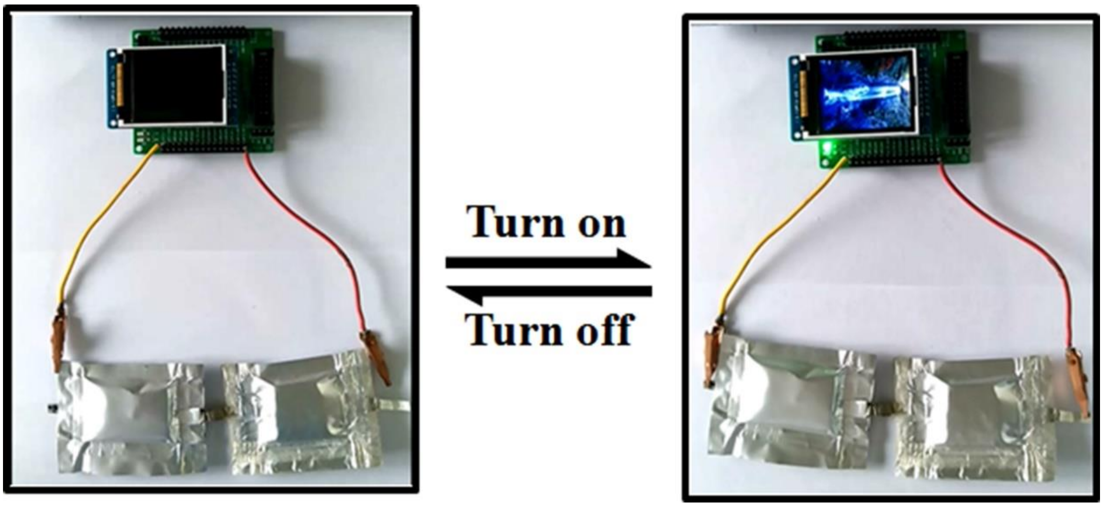

Supplementary Figure 18 | Photographs of two capacitive pouches integrated to power display. Guan Wu is the creator of the waterfall photo in the powered electronic device.

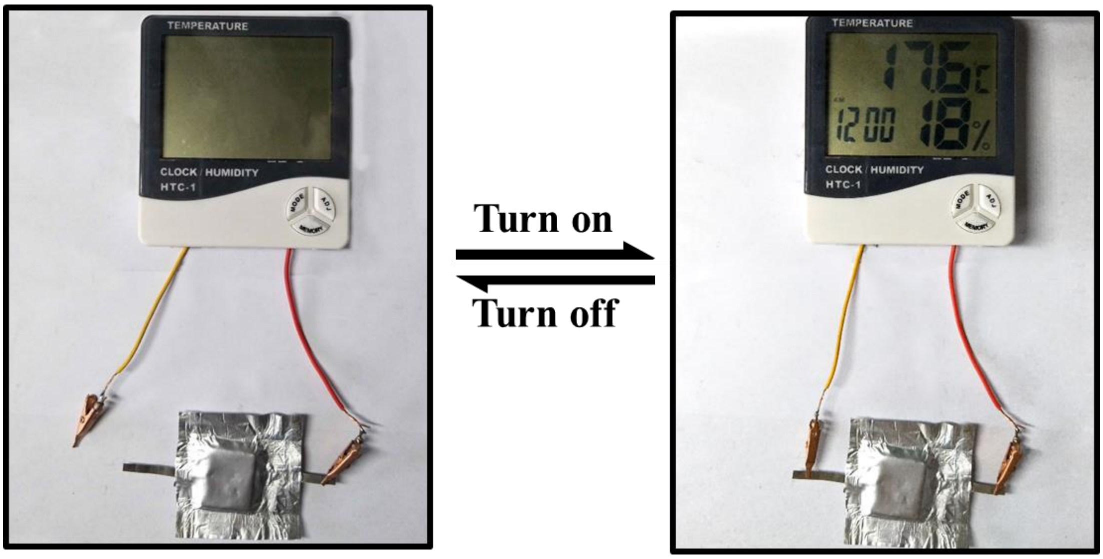

Supplementary Figure 19 | Photographs of one capacitive pouch powering monochrome display.

Supplementary References

1 Sun, G. *et al.* Hybrid Fibers Made of Molybdenum Disulfide, Reduced Graphene Oxide, and Multi-Walled Carbon Nanotubes for Solid-State, Flexible, Asymmetric Supercapacitors. *Angewandte Chemie-International Edition* **54**, 4651-4656 (2015).

2 Chen, B., Jiang, Y., Tang, X., Pan, Y. & Hu, S. Fully Packaged Carbon Nanotube Supercapacitors by Direct Ink Writing on Flexible Substrates. *Acs Applied Materials & Interfaces* **9**, 28433-28440 (2017).

3 Wu, Z.-S., Parvez, K., Feng, X. & Muellen, K. Graphene-based in-plane micro-supercapacitors with high power and energy densities. *Nature Communications* **4**, 2487 (2013).

4 Hao, C. *et al.* Flexible All-Solid-State Supercapacitors based on Liquid-Exfoliated Black-Phosphorus Nanoflakes. *Advanced Materials* **28**, 3194-3201 (2016).

5 Yu, D. *et al.* Scalable synthesis of hierarchically structured carbon nanotube-graphene fibres for capacitive energy storage. *Nat Nanotechnol* **9**, 555-562 (2014).

6 Huang, Z. *et al*. High Mass Loading MnO_2 with Hierarchical Nanostructures for Supercapacitors. *ACS Nano* **12**, 3557−3567 (2018).

7 Yao, L. *et al.* Scalable 2D Hierarchical Porous Carbon Nanosheets for Flexible Supercapacitors with Ultrahigh Energy Density. *Advanced Materials* **30**, 1706054 (2018).

8 Li, P. *et al*. Stretchable All-Gel-State Fiber-Shaped Supercapacitors Enabled by Macromolecularly Interconnected 3D Graphene/Nanostructured Conductive Polymer Hydrogels. *Advanced Materials* **30**, 1800124 (2018).

9 Pang, H. *et al.* One-pot synthesis of heterogeneous Co_3O_4-nanocube/ $Co(OH)_2$-nanosheet hybrids for high-performance flexible asymmetric all-solid-state supercapacitors. *Nano Energy* **35**, 138-145 (2017).

10 Lin, Y., Gao, Y. & Fan, Z. Printable Fabrication of Nanocoral-Structured Electrodes for High-Performance Flexible and Planar Supercapacitor with Artistic Design. *Advanced Materials* **29**, 201701736 (2017).

11 Yan, J. *et al.* Flexible MXene/Graphene Films for Ultrafast Supercapacitors with Outstanding Volumetric Capacitance. *Advanced Functional Materials* **27**, 1701264 (2017).

12 El-Kady, M. F., Strong, V., Dubin, S. & Kaner, R. B. Laser Scribing of High-Performance and Flexible Graphene-Based Electrochemical Capacitors. *Science* **335**, 1326-1330 (2012).
