# Supplementary material for: Microfluidic-spinning construction of black-phosphorus-hybrid microfibres for non-woven fabrics toward a high energy density flexible supercapacitor
Source: Nat Commun. 2018 Nov 1;9:4573. doi: 10.1038/s41467-018-06914-7 (PMC6212570; doi:10.1038/s41467-018-06914-7)
Supplement: Supplementary file 3 — Description of Additional Supplementary Files [file 41467_2018_6914_MOESM3_ESM.pdf]

Description of Additional Supplementary Files:

Supplementary Movie 1. Flexible supercapacitor stably powers LED with bending stability.

Supplementary Movie 2. Supercapacitor powers smart watch.

Supplementary Movie 3. Two capacitive pouches integrate in series to power multi-color displays. Guan Wu is the creator of the waterfall photo in the powered electronic device.

Supplementary Movie 4. Single capacitive pouch powers monochrome display.
